# Molecular Basis of ABHD5 Lipolysis Activation

**DOI:** 10.1038/srep42589

**Published:** 2017-02-17

**Authors:** Matthew A. Sanders, Huamei Zhang, Ljiljana Mladenovic, Yan Yuan Tseng, James G. Granneman

**Affiliations:** 1 Center for Integrative Metabolic and Endocrine Research Wayne State University School of Medicine, Detroit, MI 48201, USA; 2 Center for Molecular Medicine and Genetics, Wayne State University School of Medicine, Detroit, MI 48201, USA.

## Abstract

Alpha-beta hydrolase domain-containing 5 (ABHD5), the defective gene in human Chanarin-Dorfman syndrome, is a highly conserved regulator of adipose triglyceride lipase (ATGL)-mediated lipolysis that plays important roles in metabolism, tumor progression, viral replication, and skin barrier formation. The structural determinants of ABHD5 lipolysis activation, however, are unknown. We performed comparative evolutionary analysis and structural modeling of ABHD5 and ABHD4, a functionally distinct paralog that diverged from ABHD5 ~500 million years ago, to identify determinants of ABHD5 lipolysis activation. Two highly conserved ABHD5 amino acids (R299 and G328) enabled ABHD4 (ABHD4 N303R/S332G) to activate ATGL in Cos7 cells, brown adipocytes, and artificial lipid droplets. The corresponding ABHD5 mutations (ABHD5 R299N and ABHD5 G328S) selectively disrupted lipolysis without affecting ATGL lipid droplet translocation or ABHD5 interactions with perilipin proteins and ABHD5 ligands, demonstrating that ABHD5 lipase activation could be dissociated from its other functions. Structural modeling placed ABHD5 R299/G328 and R303/G332 from gain-of-function ABHD4 in close proximity on the ABHD protein surface, indicating they form part of a novel functional surface required for lipase activation. These data demonstrate distinct ABHD5 functional properties and provide new insights into the functional evolution of ABHD family members and the structural basis of lipase regulation.

The mobilization of free fatty acids (FFA) from stored triglyceride is a fundamental cellular process that is mediated in many tissues by the functional interaction of alpha-beta hydrolase domain-containing 5 (ABHD5) with adipose triglyceride lipase (ATGL). ABHD5 null mutations disrupt lipolysis and lead to ectopic lipid accumulation in *Arabidopsis*^1^, *C. elegans*^2^,^3^, mice^4^,^5^, and humans (Chanarin-Dorfman syndrome^6^). ABHD5 is essential for ATGL activation in fat and muscle where it integrates extracellular and intracellular signals in the control of lipolysis^7^,^8^,^9^. Additionally, lipase activation by ABHD5 suppresses colon cancer progression^10^,^11^ and promotes hepatitis C viral replication^12^.

The structural determinants of ABHD5 activation of ATGL are poorly understood. Although ABHD5 is a member of the alpha-beta hydrolase family, it lacks the serine nucleophile of the consensus catalytic triad, and thus lacks hydrolytic activity^8^. In adipocytes, the lipase-activating function of ABHD5 is repressed by its binding to perilipin 1 (PLIN1), a lipid droplet (LD) scaffold. Extracellular signals that activate protein kinase A (PKA) lead to phosphorylation of PLIN1 and ABHD5 and stimulate release of ABHD5, which then activates ATGL^7^,^13^,^14^. Furthermore, ABHD5 is the direct target of endogenous and synthetic ligands that modulate its lipase-activating function by regulating its interactions with inhibitory PLIN proteins^9^. Although ABHD5 binds ATGL^8^,^15^ and PLIN proteins indirectly regulate that interaction^13^,^16^,^17^,^18^, the molecular basis of ATGL activation by ABHD5 remains unclear.

To gain insights into the mechanism of ATGL activation, we performed comparative structural and evolutionary analysis of ABHD5 and ABHD4, a functionally-distinct paralog present in mammals and bony fishes that shares 50–55% sequence identity with ABHD5. ABHD4 hydrolyzes n-acyl phosphatidylserine and n-acyl phosphatidylethanolamine and does not promote ATGL activity^19^,^20^. We identified two highly conserved ABHD5 amino acids (R299 and G328) that are necessary for ATGL activation by ABHD5 and sufficient to enable that activity in ABHD4 (ABHD4 N303R/S332G). Importantly, analysis of lipolysis-inactive ABHD5 mutants demonstrated that ATGL activation was dissociable from ATGL translocation to the LD surface and from ABHD5 interactions with PLIN proteins and synthetic ABHD5 ligands. Structural modeling based on shape analysis identified a novel functional surface in ABHD5 and gain-of-function ABHD4 that contains the residues critical for ATGL activation.

## Results

### ABHD4 localizes to LDs but does not activate ATGL

**Figure 1:**
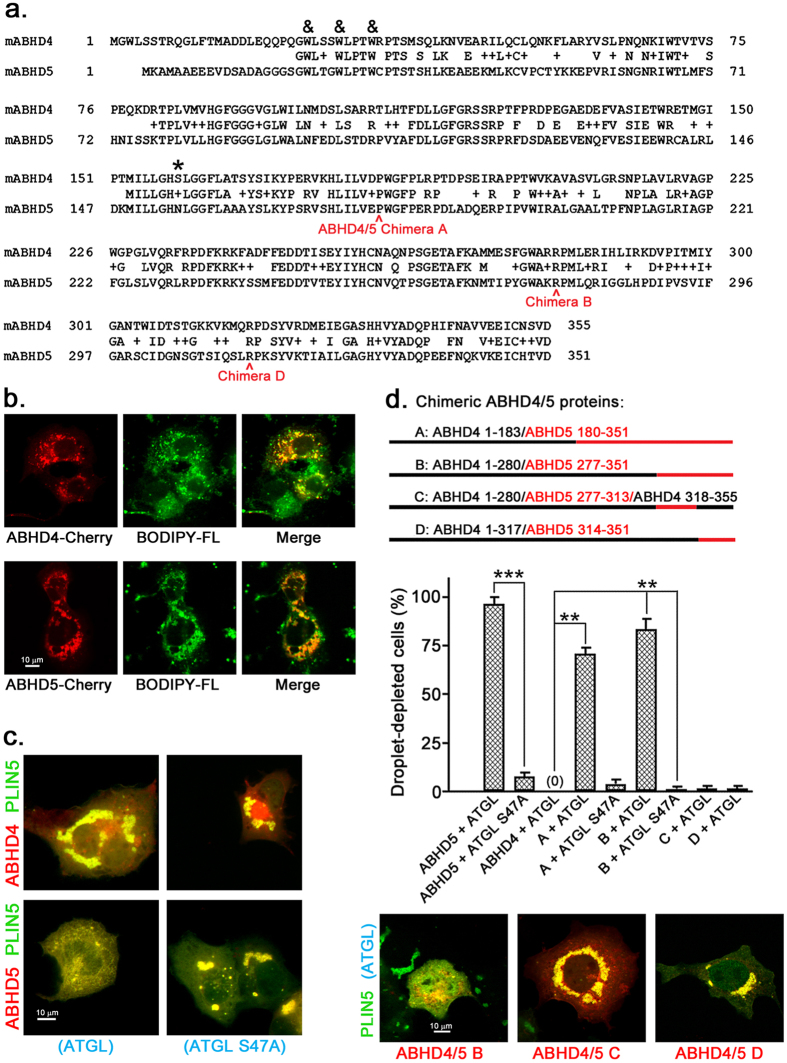
Chimeric ABHD4 1–280/ABHD5 277–351 activates ATGL-dependent lipolysis. (**a**) Sequence comparison of mouse ABHD4 and ABHD5. & indicates common tryptophans required for LD localization of ABHD5, while the active site serine present in ABHD4 but not ABHD5 is indicated by *. In this figure and subsequent figures, ABHD proteins are from mouse and numbering of amino acids refers to the mouse protein unless indicated otherwise. PLIN5 and ATGL are also from mouse unless indicated otherwise. (**b**) ABHD4 associates with LDs. Cos7 cells transfected with ABHD4-mCherry or ABHD5-mCherry were lipid loaded overnight then stained with LD marker BODIPY-Fluorescein. This experiment was performed twice with the same results. (**c**) ABHD4 does not activate lipolysis. Cos7 cells transfected with ABHD protein-mCherry, PLIN5-EYFP, and ATGL-CFP or ATGL S47A-CFP (lipase inactive) were lipid loaded overnight then fixed. This experiment was repeated more than three times with the same results. In this experiment and subsequent experiments, Cos7 cells were co-transfected with PLIN5-EYFP to mark LDs and facilitate their formation, as described previously^16^. (**d**) Analysis of chimeric ABHD4/ABHD5 protein lipolysis activation. In this experiment and all subsequent experiments utilizing this assay (Figs 2, 3 and 7 and S1), only cells visibly expressing all three proteins (ABHD protein-mCherry, PLIN5-EYFP, and ATGL-CFP or ATGL S47A-CFP (lipase inactive)) were scored by an observer blinded to the transfection conditions. In (**b**–**d**), scale bars = 10 μm. Values are means ± SEM from three independent experiments. **p < 0.01; ***p < 0.001.

In mouse, ABHD5 shares 52.4% sequence identity (184/351) with its closest paralog ABHD4 (Fig. 1a), and ABHD5 and ABHD4 paralogs in other vertebrate species have a similar sequence identity. This suggests they may have similar three-dimensional structures. Like ABHD5, ABHD4 has three highly conserved tryptophans in its N-terminal region required for ABHD5 LD localization^21^,^22^. In the absence of ATGL or PLIN proteins, ABHD4 and ABHD5 displayed similar partial localization to LDs in transfected lipid-loaded Cos7 cells, as indicated by their co-localization with the LD marker BODIPY (Fig. 1b). In cells cotransfected with ATGL, ABHD5 strongly activated lipolysis, whereas ABHD4 was inactive (Fig. 1c). Since ABHD4 is able to localize to LDs, this indicates its inability to activate ATGL is not due to differences in cellular localization from ABHD5.

### The C-terminal 75 amino acids of ABHD5 are critical for ATGL activation

We next examined the ability of chimeric ABHD4/ABHD5 proteins to activate ATGL in Cos7 cells (Fig. 1d). Since previous work indicated ABHD5 C-terminal deletion mutants do not activate ATGL^22^, we tested chimeras containing decreasing amounts of the ABHD5 C-terminal region. An ABHD4/5 chimera containing only the 75 C-terminal amino acids of ABHD5 (chimera B; 4 1–280/5 277–351) activated lipolysis in Cos7 cells similarly to wild-type ABHD5. Chimeras containing either half of this ABHD5 region (4 1–280/5 277–313/4 318–355 (chimera C) and 4 1–317/5 314–351 (chimera D)), however, were inactive, indicating amino acids within both ABHD5 277–313 and 314–351 are necessary for ATGL activation (Fig. 1d).

### Identification of ABHD5 residues required for ATGL activation

**Figure 2:**
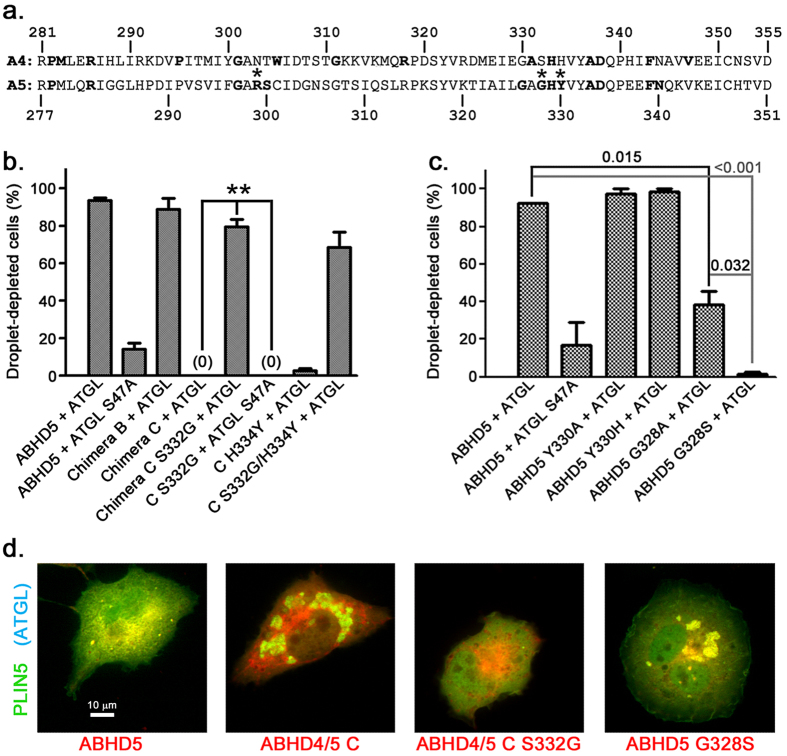
ABHD5 G328 is essential for ATGL activation. (**a**) Homology analysis of vertebrate ABHD4 and ABHD5 paralogs. Identical residues in all vertebrate homologs of each protein are indicated in bold, based on analysis of proteins in the NCBI database as of May 2016. Asterisks indicate ABHD5 identical residues not conserved between ABHD4 and ABHD5. (**b**) Chimera C S332G activates lipolysis in Cos7 cells. **p < 0.01. (**c**) ABHD5 G328S does not activate lipolysis. Values are means ± SEM from three independent experiments. (**d**) Representative images of ABHD mutant transfected Cos7 cells. Scale bar = 10 μm.

We reasoned that amino acids required for ATGL activation would be highly conserved among vertebrate ABHD5 homologs but not conserved between ABHD4 and ABHD5. We identified 13 amino acids within ABHD5 277–351 that were identical in all vertebrate ABHD5 homologs (Fig. 2a). Of these 13 amino acids, three were not conserved with ABHD4: R299, within ABHD5 277–314, and G328 and Y330, within ABHD5 315–351.

### ABHD5 G328 is critical for ATGL activation

We tested whether ABHD5 amino acids identified by homology analysis could confer on ABHD4 the ability to activate ATGL. In Cos7 cells, chimera C S332G activated lipolysis similarly to both full length ABHD5 and chimera B containing the entire 75 C-terminal ABHD5 amino acids. Chimera C H334Y, however, did not activate lipolysis (Fig. 2b and d). Reciprocal mutation of ABHD5 G328 to alanine or the corresponding highly conserved vertebrate ABHD4 serine inhibited or abolished, respectively, activation, while ABHD5 Y330H was fully active (Fig. 2c and d).

### ABHD4 N303R/S332G activates ATGL

**Figure 3:**
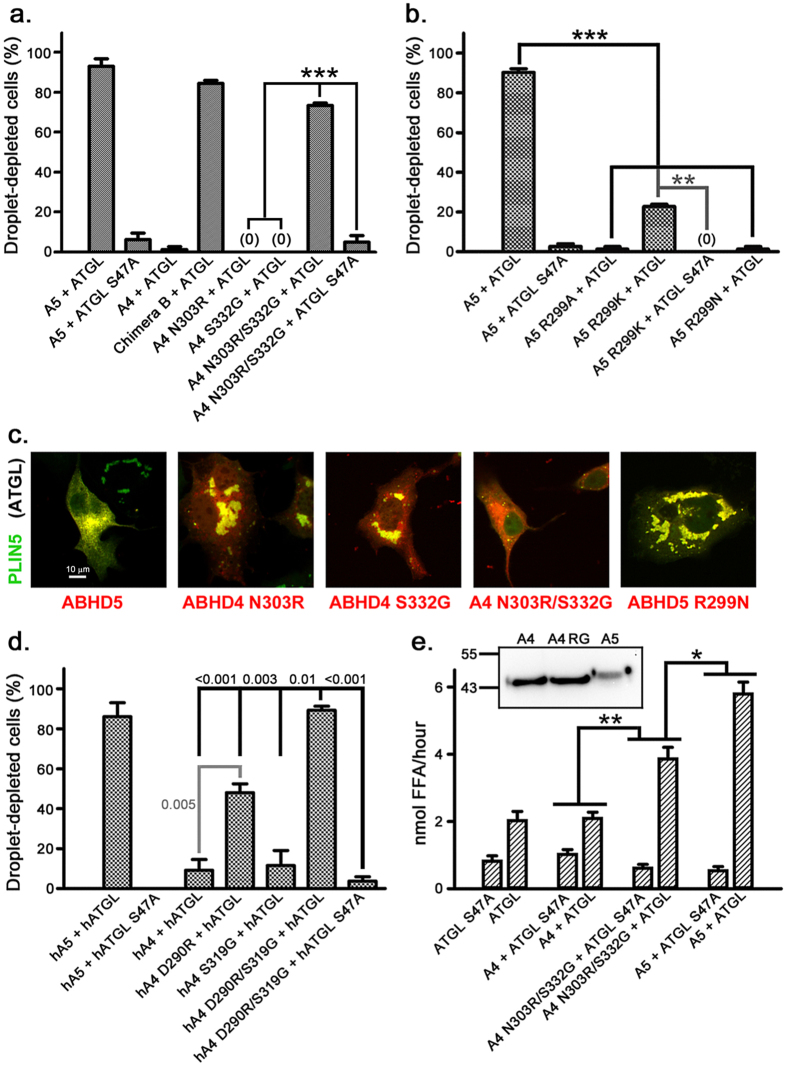
ABHD5 R299 is essential for ATGL activation. (**a**) ABHD4 N303R/S332G activates ATGL-dependent lipolysis in transfected Cos7 cells. (**b**) ABHD5 R299N does not activate lipolysis. (**c**) Representative images of ABHD mutant transfected Cos7 cells. Scale bar = 10 μm. (**d**) Human ABHD4 D290R/S319G activates lipolysis. (**e**) Partially purified ABHD4 N303R/S332G activates lipolysis in artificial LDs. Inset shows strep tag immunoblot of relative ABHD protein levels in assay. For (**a**,**b**,**d**,**e**), values are means ± SEM from three independent experiments. *p < 0.05; **p < 0.01; ***p < 0.001.

Because ABHD4/5 chimera C and chimera D (Fig. 1c) are both inactive, this indicates that amino acids within both ABHD5 277–313 and ABHD5 315–351 appear to be critical for ATGL activation. Having determined that chimera C S332G is active, we sought to identify amino acids within ABHD5 277–313 that are necessary for ATGL activation. R299 is the only amino acid in this region identical in all vertebrate ABHD5 homologs but not present in mouse ABHD4 or any of its vertebrate homologs (Fig. 2a). While single ABHD4 reciprocal mutations (N303R or S332G) did not confer activity by themselves, ABHD4 N303R/S332G activated ATGL-dependent lipolysis similarly to wild type ABHD5 in transfected COS7 cells (Fig. 3a and c). Mouse ABHD5 with the reciprocal R299N mutation (Fig. 3b and c) and ABHD5 R299A (Fig. 3b) were completely inactive, while the conservative R299K mutation strongly attenuated activity (Fig. 3b). We confirmed the importance of these amino acids in ATGL activation using human ABHD4 with the corresponding mutations (hABHD4 D290R/S319G) and human ATGL. ATGL-dependent lipolysis activation by hABHD4 D290R/S319G and full-length hABHD5 was the same in COS7 cells, and, unlike the corresponding mouse protein, the single mutant hABHD4 D290R displayed partial activity (Fig. 3d). Thus, although ABHD4 and ABHD5 paralogs diverged and began evolving distinct functions more than 450 million years ago^23^,^24^,^25^, a two amino acid change is sufficient to confer on ABHD4 the ability to activate ATGL.

### ABHD4 N303R/S332G activation of ATGL does not require ABHD4 hydrolase activity

As noted above, ABHD5 lacks the consensus serine nucleophile present in ABHD4 (S159), and does not have hydrolase activity. It is thus possible that activation of ATGL-dependent lipolysis by ABHD4 N303R/S332G requires ABHD4 hydrolase activity. Triple mutant ABHD4 S159A/N303R/S332G, however, activated lipolysis similarly to ABHD4 N303R/S332G in transfected Cos7 cells, indicating that ATGL activation by ABHD4 N303R/S332G is independent of ABHD4 hydrolase activity (Figure S1).

### ABHD4 N303R/S332G activates ATGL-dependent lipolysis on artificial LDs

Although the Cos7 LD assay provides a sensitive readout indicating that ABHD4 N303R/S332G activated lipolysis, it might not precisely gauge its activity relative to ABHD5. We therefore examined lipolysis activation in an *in vitro* assay consisting of partially purified ABHD proteins, lysates from ATGL or ATGL S47A transfected Cos7 cells, and artificial LDs^26^. We found that ABHD4 N303R/S332G significantly stimulated ATGL-dependent lipolysis compared to ABHD4, which was inactive compared to ATGL lysate alone, though lipolysis activation was less than that observed with partially-purified ABHD5 (Fig. 3e).

### Characterization of ABHD5 loss-of-function and ABHD4 gain-of-function mutants in brown adipocytes

**Figure 4:**
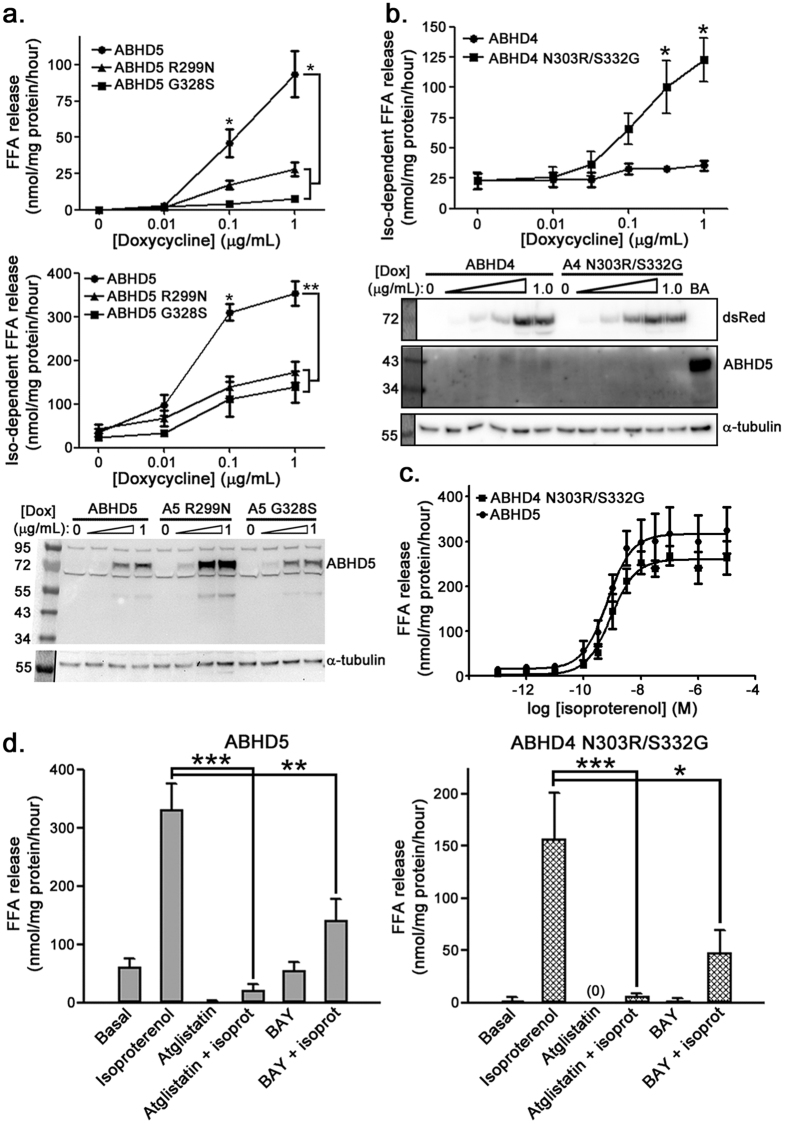
Characterization of loss-of-function and gain-of-function ABHD proteins in ABHD5-null BAs. Doxycycline-inducible ABHD-mCherry proteins were expressed in a BA cell line in which endogenous ABHD5 was silenced by shRNA. In the immunoblots shown in 4a and 4b, it should be noted that the mCherry tag shifts the ABHD protein molecular weight from ~43 kDa for endogenous ABHD5 to ~72 kDa for the mCherry-tagged protein. (**a**) ABHD5 R299N and G328S mutations inhibit basal (top panel) and isoproterenol-stimulated lipolysis (middle panel). Values are mean ± SEM from three or more independent experiments. Representative ABHD5 immunoblot is shown below, demonstrating absence of endogenous ABHD5 (~43 kDa) in all cell lines. ***p < 0.05 or < 0.01, respectively, compared to ABHD5 mutant cell lines. (**b**) Lipolysis activation in BA cell lines expressing ABHD4 or ABHD4 N303R/S332G. Representative dsRed (ABHD protein mCherry tag) immunoblot is shown below. Values are means ± SEM from four independent experiments. BA, lysate from differentiated wild-type BA cells. *p < 0.05 compared to ABHD4. (**c**) Isoproterenol dose-response of BA cell lines expressing ABHD5 (A5) or ABHD4 N303R/S332G (A4 RG). In order to obtain similar levels of ABHD4 protein expression (Figure S2b), ABHD5 and ABHD4 N303R/S332G BA cells were induced with, respectively, 0.0125 and 1.0 μg/mL doxycycline for 42–48 hrs. Mean ± SEM from four independent experiments is shown. (**d**) ABHD5 and ABHD4 N303R/S332G BA cell line lipolysis requires ATGL and hormone sensitive lipase. Cells were treated with 10 μM atglistatin or 5 μM BAY 59–9435 in the presence or absence of 100 nM isoproterenol. In 4d, expression of ABHD protein in both cell lines was induced with 1 μg/mL doxycycline, which induces higher expression of ABHD5 than ABHD4 N303R/S332G (Figure S2a). Mean ± SEM from three independent experiments is shown. *p < 0.05; **p < 0.01; ***p < 0.001.

To characterize activity in a more physiologically relevant cell system, we expressed ABHD5 loss-of-function mutants and the ABHD4 N303R/S332G gain-of-function mutant in a brown adipocyte (BA) cell line in which endogenous ABHD5 expression was silenced by viral shRNA^9^. At 1 μM doxycycline, ABHD5 re-expression increased basal and isoproterenol-stimulated lipolysis by >70- and 10-fold, respectively, compared to lipolysis in the absence of ABHD5 (i.e., no doxycycline). Lipolysis in ABHD5 S332G or ABHD5 R299N BA cells was strongly reduced compared to ABHD5 (Fig. 4a). Isoproterenol significantly stimulated lipolysis in ABHD4 N303R/S332G BA cells (Fig. 4b) compared to BA cells expressing similar levels of ABHD4, which did not respond to isoproterenol. To control for higher doxycycline-induced protein expression of ABHD5 compared to ABHD4 N303R/S332G (Figure S2a), we titrated the doxycycline concentration used to induce ABHD5 to yield more equal levels of expression (Figure S2b). Under these conditions we found that isoproterenol stimulated FFA release with similar potency (ABHD5 EC_**50**_, 0.668 ± 0.164 nM; ABHD4 N303R/S332G EC_**50**_, 0.895 ± 0.272 nM) and efficacy in each cell line (ABHD5 response, 300.9 ± 17.2 nmol/hr/mg protein FFA; ABHD4 N303R/S332G response, 256.9 ± 18.7 nmol/hr/mg protein FFA; Fig. 4c). As observed for ABHD5, lipolysis in ABHD4 N303R/S332G BA cells was completely inhibited by the ATGL inhibitor atglistatin^27^. Additionally, BAY 59–9435 (BAY)^28^, a selective inhibitor of the diglyceride lipase hormone-sensitive lipase that is downstream of ATGL, similarly inhibited lipolysis activation by ABHD5 and ABHD4 N303R/S332G (Fig. 4d). These data further confirm that ABHD4 N303R/S332G and ABHD5 engage the same endogenous lipases in BAs.

### ABHD5 loss-of-function mutations do not affect ABHD5 interactions with PLIN proteins or binding of ABHD5 ligand SR4995

**Figure 5:**
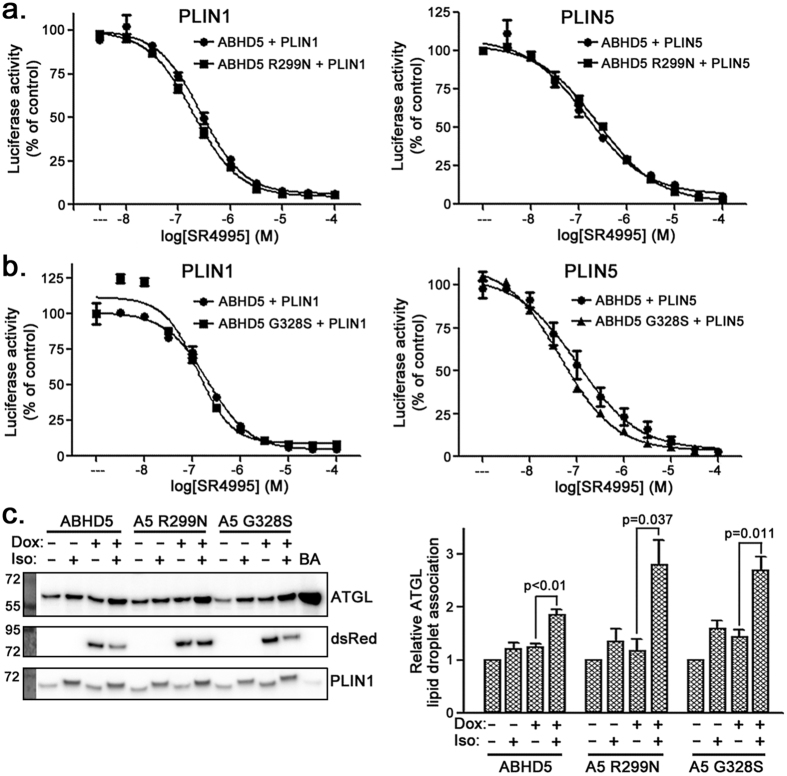
Effect of ABHD5 loss-of-function mutations on ligand binding, association with PLIN proteins, and ATGL translocation. (**a**,**b**) ABHD5 loss-of-function mutations do not affect ligand binding or association with PLIN proteins. Mean ± SD from one of at least two independent experiments with similar results is shown. (**c**) ABHD5 loss-of-function mutations do not affect ATGL translocation to LDs in BA cells. LDs were isolated from the indicated differentiated BA cell lines that were treated with 100 nM isoproterenol (or untreated control cells) for 30 mins. Densitometric analysis (mean ± SEM) in right panel is based on three independent experiments.

ABHD5 G328S and R299N associated with PLIN5 on LDs (Figs 2d and 3c), suggesting that disruption of ATGL activation caused by these mutations did not result from general disruption of ABHD5 structure and function. We further examined the effect of these mutations on ABHD5 interactions with PLIN proteins and ABHD5 ligand binding using protein complementation analysis. ABHD5 ligand SR4995 binding dissociates ABHD5 from PLIN1 or PLIN5, leading to ABHD5 activation of lipolysis^9^. SR4995 disrupted interactions of ABHD5, ABHD5 R299N (Fig. 5a), and ABHD5 G328S (Fig. 5b) with PLIN proteins at similar potency. These results demonstrate that R299N and G328S mutations selectively disrupt ABHD5 activation of ATGL without affecting ABHD5 interaction with PLIN proteins or its ligand SR4995.

### ABHD5 loss-of-function mutations do not affect ATGL trafficking in live BAs

ABHD5 and ATGL directly interact in biochemical assays^8^,^15^, and ATGL translocates to LDs in response to PKA activation^7^,^13^. We examined PKA-induced translocation of ATGL in BA cells lacking ABHD5 and in those expressing wild type or loss-of-function mutants R299N and G328S. In all cell lines, ATGL was partially localized on LDs in the basal state and isoproterenol triggered significant translocation to LDs (Fig. 5c). Thus, while isoproterenol-dependent translocation was highly dependent on the presence of ABHD5 protein (i.e., doxycycline-induced), lipolysis-inactivating mutants of ABHD5 did not affect this process.

### Structural models of ABHD5 and ABHD4

**Figure 6:**
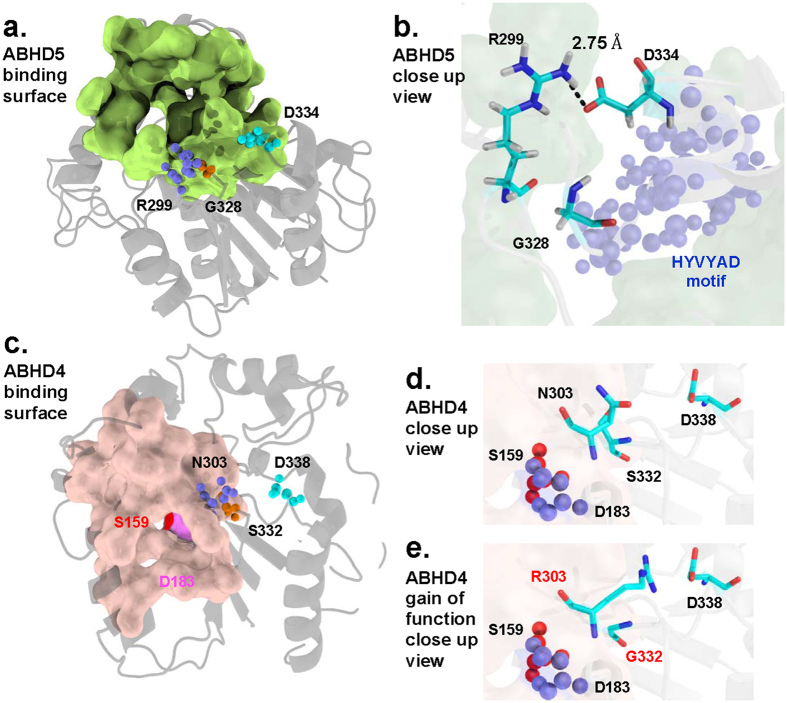
Shape analysis of ABHD5 and ABHD4 binding surfaces. (**a**) The functionally important residues D334 (cyan), R299 (slate), and G328 (orange) in spheres are spatially clustered on the predicted ABHD5 binding surface (light green). (**b**) A close up view shows that residues G328 and D334 are centered around R299. The model predicts the local structure of ABHD5 may be stabilized by a salt-bridge between the D334 carboxyl group (red) and the R299 guanidinium group (blue). The HYVYAD motif containing D334 is shown in blue spheres. (**c**) The predicted catalytic ABHD4 residues D183 (violet) and nucleophile S159 (red) form a dyad located inside the deep tunnel of the identified ABHD4 binding surface (light orange). ABHD4 N303 (slate), S332 (orange) and D338 (cyan) are spatially oriented similarly to the corresponding key residues R299, G328, and D334 in ABHD5 (**a**) and are situated away from the ABHD4 catalytic center S159. (**d**) Close up view of ABHD4 N303, S332, and D338. Note that the region occupied by N303, S332 and D338 is spatially remote from the ABHD4 catalytic residues S159 and D183 (spheres). (**e**) In ABHD4 N303R/S332G, R303, G332 and D338 are spatially oriented similarly to R299, G328, and D334 in ABHD5 (**b**), conferring on ABHD4 N303R/S332G the ability to activate ATGL.

Large-scale geometric analysis indicates that functionally important amino acids tend to be spatially clustered on a binding surface^29^,^30^,^31^,^32^. To identify functionally important surfaces and interpret our mutational analyses we built topology-based computational models^33^,^34^,^35^ of ABHD4 and ABHD5 (see Methods). Initial ABHD5 and ABHD4 models were generated using multiple related 3D structures and homology modeling and then refined using shape analysis with molecular dynamic (MD) simulations (Fig. 6). The overall scaffolds of ABHD5 and ABHD4 models have highly similar topologies of alpha helices and beta sheets with multiple loops bridging them together. ABHD5 geometric analysis identified a putative binding surface on ABHD5 consisting of 63 pocket residues with a mean molecular volume of 2360.73 Å^3^ and a solvent accessible area of 1514.43 Å^2^. This predicted binding surface has a functional pocket that can accommodate endogenous ligands such as acyl-CoA or synthetic compounds (e.g., SR4995) that regulate ABHD5 interactions with perilipin proteins^13^,^16^,^36^. Within this binding pocket, our shape analysis further identified the functionally important residues R299 and G328 demonstrated experimentally to be critical for ATGL activation. Using R299 as a geometric center, we calculated a profile of pair-wise atomic interactions in the 3D Alpha Shape Delaunay triangulation (Table 1) based on the refined ABHD5 models. Spatial neighbors of R299 included T246, G328, Y330, and D334 (Fig. 6a and b). In particular, the close proximity between *Nη*_2_ of R299 and *Oδ*_2_ of D334 (*Nη*_2_ − *Oδ*_2_ 2.75 Å) indicated a salt bridge may maintain the binding surface shape required for ATGL activation. The ABHD5 trajectory in MD simulations further revealed the fluctuating distance in a range of 13.8 Å between *N*η_2_ of R299 and Oδ_2_ of D334, which indicates that ABHD5 may undergo large conformational changes when it activates ATGL.

**Figure 7:**
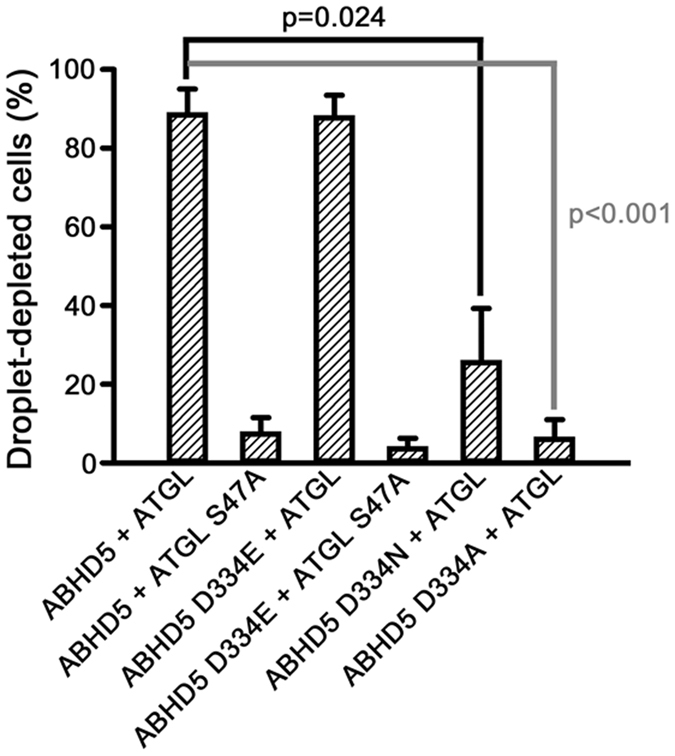
ABHD5 lipolysis activation requires a negatively charged amino acid at position 334. Cos7 cells were transfected with wild-type ABHD5 or the indicated ABHD5 D334 mutant constructs then lipid loaded and scored for lipid droplet content by blinded analysis. Results are mean ± SEM from three independent experiments.

Geometric modeling indicated ABHD4 contains a binding surface and relative distance among key amino acids very similar to ABHD5 (Fig. 6c). However, the corresponding ABHD4 surface (Fig. 6d) differs by the presence of N303 and S332, which disable lipase activation in ABHD5 mutants. Our MD analysis of gain-of-function mutant ABHD4 N303R/S332G (Fig. 6e) indicated R303, G332, and D338 in this protein shared structural similarity with ABHD5 R299, G328 and D334. ABHD4 has phospholipase B activity and it should be noted that the S159 hydroxyl group at the ABHD4 catalytic center (*O*_*r*_) is spatially remote (>16.5 Å) from D338 and the N303R and S332G mutation sites. Consistent with the model prediction placing ABHD5 D334 in close proximity to R299 and G328, D334 mutation to alanine or asparagine strongly inhibited ABHD5 lipolysis activation in Cos7 cells. The charge-preserving mutation D334E, however, preserved activity, indicating activation requires a negatively charged amino acid at this position (Fig. 7).

## Discussion

Intracellular lipolysis is a critical metabolic process of virtually all eukaryotic cells, and tight control is important to metabolic health. ABHD5 is a critical regulator of ATGL activity that is capable of integrating extracellular and intracellular signals, but the mechanism by which ABHD5 activates ATGL is unclear. We employed comparative evolutionary analysis of ABHD5 and its functionally distinct paralog ABHD4 to identify specific amino acids in mammalian ABHD5 required for ATGL activation. Importantly, stimulation-dependent ATGL translocation to LDs was not suppressed by ABHD5 loss-of-function mutations. Thus, ABHD5-dependent translocation of ATGL to LDs is not sufficient to account for the ABHD5 lipase activating function. Furthermore, ABHD5 loss-of-function mutations R299N and G328S did not affect PLIN interactions or allosteric regulation by synthetic ligands, demonstrating ABHD5 lipolysis activation is distinct from its other functions.

Mutational analysis indicated R299 and G328, which are unique to ABHD5, and D334, which is conserved in all vertebrate ABHD4/5 members, play critical roles in lipase activation. R299 is identical in all vertebrate ABHD5 homologs sequenced to date, and the fact that D290R substitution in human ABHD4 alone was sufficient to allow partial activation, whereas the conservative R299K substitution in mouse ABHD5 was highly defective, indicates R299 has a positive and essential role in ATGL activation. R299 is predicted by our structural models to be close to G328, which is also identical in all vertebrate ABHD5 homologs. Although ABHD5 G328S is highly defective, the ABHD5 G328A mutant retained significant (~40% of ABHD5) activity, indicating that glycine in this position is not absolutely essential for lipase activation. Nonetheless, in gain-of-function mutant ABHD4 N303R/S332G, R303 and G332 strongly synergize to activate lipolysis. The mechanism for this synergy is not known, but may involve specific interactions with the LD phospholipid monolayer or, more speculatively, enzymatic activity.

Our ABHD5 structural models placed R299 at the ABHD5 surface, where it could interact with either the LD surface or ATGL. Arginine is thought to have a strong capacity for creating perturbations in phospholipid membranes because its high pKa allows it to remain protonated and it has five potential H-bond donors available for interactions with water and phospholipid head groups^37^. In contrast, lysine, which only weakly promotes lipolysis when substituted for R299 (Fig. 3b), is theorized to be less capable of creating membrane perturbations owing to its lower pKa and fewer potential H-bond donors. Interestingly, a group of three highly conserved basic amino acids (ABHD5 RKR 235–237) that is also present in ABHD4 is required for ABHD5-mediated hepatitis C virus production and lipolysis in Lunet N hCD81 cells^12^. It is possible R299 acts in concert with other basic amino acids in ABHD5 to create the LD membrane perturbation necessary for ATGL access to triglycerides. In this regard, the G328 amide backbone may interact with membrane phospholipid to allow favorable interactions of R299 and D334. It seems less likely that R299 participates directly in ATGL-mediated hydrolysis, since the high pKA of arginine makes it a poor candidate for serving as a general base in a hydrolase catalytic reaction.

ABHD5 R299 is close to D334 in our structural models, and mutagenesis analysis indicates a negatively-charged amino acid at 334 is necessary for ATGL activation. While it is possible a negatively charged amino acid interacts with the choline head group of phosphatidylcholine on the LD surface, D334 mutation to A or N might also disrupt local ABHD5 structure by abolishing a possible ionic interaction between D334 and R299.

Our model predicts Y330 is also a close neighbor of R299. Interestingly, Y330 is the direct target of the covalent affinity label NBD-HP-HE, which competes with SR4995 for binding to ABHD5. Binding of SR4995 to ABHD5 rapidly disrupts its association with PLIN proteins and triggers lipolysis in fat and muscle^9^. Thus, the amino acids involved in PLIN interactions and ligand regulation are in close proximity to those involved in lipase activation, and suggest a plausible model for how ABHD5 integrates extracellular and intracellular signals to control lipolysis.

ABHD5 is most closely related to ABHD4, but whereas ABHD4 contains a serine nucleophile and has phospholipase B activity^19^,^20^, vertebrate ABHD5 homologs lack this serine nucleophile and direct lipase activity^8^. Most invertebrates contain at least one ABHD paralog equally related to mammalian ABHD4 and ABHD5, making it difficult to predict function. Our analysis of mammalian ABHD4 and 5 indicates ABHD4/5 paralogs possessing the equivalent of mouse ABHD5 R299, G328, and D334 will activate lipolysis. This assertion was recently confirmed for *C. elegans* ABHD4/5 paralog C37H5.3 3. The *C. elegans* ABHD4/5 paralog Lid1, which contains lysine at the position corresponding to ABHD5 R299, also activates lipolysis^2^. We note that many invertebrate ABHD4/5 paralogs contain R, G, and D at these positions as well as the active site serine homologous to ABHD4 S159. It will be of great interest to determine whether invertebrate ABHD4/5 paralogs such as *Drosophila melanogaster* CG1882 and *C. elegans* C37H5.3 possess dual hydrolase/lipase-activating functions.

In summary, we identified a novel ABHD5 functional surface specifically required for lipase activation independently of ATGL lipid droplet translocation or interaction with PLIN proteins and synthetic ABHD5 ligands. Evolutionary analysis and experimental analysis of informative ABHD4/5 mutants demonstrated that the distinct functional properties of ABHD5, including lipase activation, PLIN protein interaction, and allosteric ligand regulation, are independently evolved and amenable to further molecular dissection.

## Methods

### Microscopy

Images were acquired with an Olympus IX-81microscope equipped with a spinning disc confocal unit. Images were captured using a 40x or 60x 1.2 NA plan apo water immersion lens and a Hamamatsu ORCA cooled CCD camera. The following Chroma filter sets were used for the indicated fluorophores: mCherry, 41043; EYFP, 31044; ECFP, 41028. Microscope control and data acquisition were performed using IPlabs (Scanalytics, BD Biosciences) software.

### Lipid droplet scoring

Cos7 cells plated on coverslips in 12-well dishes were transfected with 0.5 μg each/well of mCherry-tagged ABHD protein, PLIN5-EYFP, and either ECFP-tagged ATGL or ATGL S47A (lipase inactive) using Lipofectamine^®^ and Plus reagent^®^ (Invitrogen) as described by the manufacturer. Cells were then lipid loaded for 16–20 hrs with 200 μM oleic acid, then fixed with 4% paraformaldehyde. LD scoring was performed by an investigator blinded to transfection conditions. For each transfection condition in each experiment 25 or more cells visibly expressing all three proteins were scored. Mutant ABHD proteins were made using standard molecular biological methods, and all PCR-derived proteins were confirmed by sequencing. ABHD proteins are from mouse and numbering of amino acids refers to the mouse protein unless indicated otherwise. PLIN5 and ATGL are also from mouse unless indicated otherwise.

### Stable knockdown of ABHD5 and inducible ABHD protein expression in brown adipocytes (BA)

The mouse BA cell line used in this work has been previously described^38^. To create doxycycline-inducible BA cell lines re-expressing ABHD5 and ABHD5 mutant proteins, we mutated the ABHD5 sequence targeted by the shRNA while maintaining the wild type ABHD5 amino acid sequence, and tagged the ABHD protein with mCherry^9^. Doxycycline-inducible expression was achieved by transferring the shRNA-resistant construct into pINDUCER20^39^, and the resulting lentivirus was used to infect the brown adipocytes in which endogenous ABHD5 was stably knocked down. Doxycycline-inducible expression of ABHD proteins was measured by mCherry fluorescence of cell lysates or by immunoblotting using a rabbit polyclonal dsRed antibody (Clontech) or an ABHD5 rabbit polyclonal antibody^16^. Rabbit polyclonal antibody to α/β-tubulin was from Cell Signaling Technology.

### Analysis of lipolysis in BA cell lines

Transfected BA cell lines were differentiated for three days as described^40^ prior to induction with the indicated [doxycycline] for 40–48 hrs. Doxycycline induction medium was then replaced with HEPES-buffered Kreb’s Ringer buffer containing 1% bovine serum albumin (KRBB) and the indicated concentrations of isoproterenol, atglistatin, or BAY 59–9435. After two hrs at 37 °C, KRBB buffer was collected and accumulated fatty acids were measured using a WAKO non-esterified fatty acid (NEFA) kit adapted to Amplex Red fluorescence detection as described^41^. Amplex Red fluorescence (excitation 545 nm/emission 600 nm) was read using a BMG Labtech Clariostar plate reader. Results are reported as nmol fatty acid efflux/mg protein/hr.

### *In vitro* lipolysis assays

For *in vitro* lipolysis measurements, Strep-His(8)-tagged ABHD4, ABHD4 N303R/S332G, and ABHD5 expressed in High-Five insect cells were partially purified by His60 Ni^2+^ Superflow Resin column chromatography (Clontech). ABHD proteins were resuspended in 100 mM potassium phosphate buffer (pH 7.0) containing artificial LDs and lysates (50 μg protein) of COS7 cells transfected with ATGL or ATGL S47A in 100 μL total volume. Artificial lipid droplets (phosphatidylcholine:phosphatidylinositol 3:1 with glycerol trioleate substrate) and cell lysates were prepared as described^26^. After two hrs at 37 °C, FFAs were measured using a WAKO NEFA kit adapted to Amplex Red fluorescence detection as described^41^. Results are reported as nmol FFAs released/hr. Immunoblots of purified proteins were detected by a mouse monoclonal strep tag antibody (Qiagen).

### Protein complementation assay (PCA)

The PCA procedure has been described^7^. Protein-matched aliquots from lysates of HEK293 cells transfected with ABHD5 (or ABHD5 mutant)-luciferase(N) or PLIN1- or PLIN5-luciferase(C) were incubated together with increasing half-log doses of ABHD5 ligand SR4995 for four hrs at room temperature, and luminescence read after adding coelenterazine substrate.

### Measurement of ATGL translocation

BA cell lines transfected with doxycycline-inducible ABHD5, ABHD5 R299N, or ABHD5 G328S were differentiated three days, then continued in differentiation medium or induced 40–48 hrs with the appropriate [doxycycline] (ABHD5, ABHD5 G328S, 1 μM; ABHD5 R299N, 0.1 μM) for similar protein expression. BA cells were then incubated in KRBB with or without 100 nM isoproterenol for 30 min at 37 °C. Cells were rinsed and harvested in 20 mM HEPES pH 7.0, 250 mM sucrose, 1 mM EDTA (HSE buffer) then lysed by passage 25x through a 26 G syringe. After centrifugation (1 min 10,000 × g), lysate below the top lipid layer was removed with a syringe and the LD layer was rinsed 3x by resuspension in HSE buffer and recentrifugation. LDs were then resuspended in RIPA buffer and analyzed by SDS-PAGE. ATGL immunoblots using a rabbit polyclonal antibody^17^ were imaged on an Azure Biosystems C600 imager and quantified using Image J software. PLIN1 immunoblots were detected using a rabbit polyclonal antibody^42^.

### Structural modeling and molecular dynamics simulations

Since neither ABHD4 or ABHD5 have experimentally determined 3D structures, we generated a computational model based on homology modeling and shape analysis. We first used psi-blast^43^ to iteratively search against PDB^44^ to obtain related ABHD protein structures as templates for homology modeling using Modeller^45^. We constructed an ABHD4 model based on soluble epoxide hydrolase (PDB1s8o, 28% sequence identity) and haloalkane dehalogenase (PDB3sk0, 26%) as a template, whereas we obtained an ABHD5 model from a template based on valacyclovir hydrolase (PDB2ocg, 18% sequence identity) and an epoxide hydrolase (PDB1qo7, 14%). Although the pair-wise sequence identity between the epoxide hydrolase template and ABHD5 is less than 15%, the folds of the selected templates from ABHD family members are highly conserved across species. Based on the CATH structural classification^46^, their core structures have been classified into the same fold of 3.40.50.1820 (Alpha Beta 3-Layer aba Sandwich). Both of our initial models for ABHD4 and ABHD5 have a GA341 score of >0.90^47^, indicating that they have high reliability of fold prediction. To further refine our initial models, we conducted MD simulations using GROMACS^48^ with ABMBER all-atom force field^49^. Simulations were conducted in explicit solvents and counter ions for 30 nsec after target proteins were solvated and equilibrated in the dynamic equilibrium state. Protein conformers (structures of a target protein at a given time) in a trajectory were then computed for shape analysis of dynamic surfaces and pocket identification.

### Shape analysis of predicted functional pockets

Refined 3D ABHD4 and ABHD5 models were analyzed by topology-based geometric calculations based on the Alpha Shape Theory^50^,^51^,^52^. Briefly, we first triangulated atomic coordinates of a refined model using the weighted Delaunay triangulation^33^. We then identified putative pockets (local surfaces) of structural models by applying the flow algorithm (http://cast.engr.uic.edu/)^33^,^53^ and the SplitPocket algorithm (http://pocket.med.wayne.edu/)^34^,^54^ to characterize local protein surfaces. For each identified pocket, we computed geometric measurements for pocket residues, solvent accessible area and molecule volume. We further extracted pocket residues and identified spatial patterns^55^ for functional analysis by mutagenesis.

### Statistical analysis

All values are reported as means ± SEM with each independent experiment counting as one replicate. All experiments were performed independently at least three times unless indicated otherwise. Experimental results were analyzed by Student’s t test and considered significant at p < 0.05.

## Additional Information

**How to cite this article:** Sanders, M. A. *et al*. Molecular Basis of ABHD5 Lipolysis Activation. *Sci. Rep.* **7**, 42589; doi: 10.1038/srep42589 (2017).

**Publisher's note:** Springer Nature remains neutral with regard to jurisdictional claims in published maps and institutional affiliations.

## Supplementary Material

Supplementary Information

## Figures and Tables

**Table 1:** The spatial distances *d(a*_1_, *a*_2_) in Å, where *a*_1_ and *a*_2_ are atoms, measured between the N atom of the G328 amide and the O atoms of the D334 carboxyl group near the guanidinium group of R299.

		G328	D334	
*d(a*_1_, *a*_2_)		*N*	*Oδ*_1_	*Oδ*_2_
R299	*Nε*	5.02	4.60	3.15
	*Nη*_1_	7.01	5.73	4.80
	*Nη*_2_	6.17	3.48	2.75
