# Supplementary material for: Molecular Basis of ABHD5 Lipolysis Activation
Source: Sci Rep. 2017 Feb 17;7:42589. doi: 10.1038/srep42589 (PMC5314347; doi:10.1038/srep42589)
Supplement: Supplementary Information [file srep42589-s1.pdf]

Molecular Basis of ABHD5 Lipolysis Activation

Matthew A. Sanders[1,2], Huamei Zhang[1,2], Ljiljana Mladenovic[1,2], Yan Yuan Tseng[2,*], and James G. Granneman[1,2,*]

From the [1]Center for Integrative Metabolic and Endocrine Research and [2]Center for Molecular Medicine and Genetics, Wayne State University School of Medicine, Detroit, MI 48201

Supplementary Figure S1

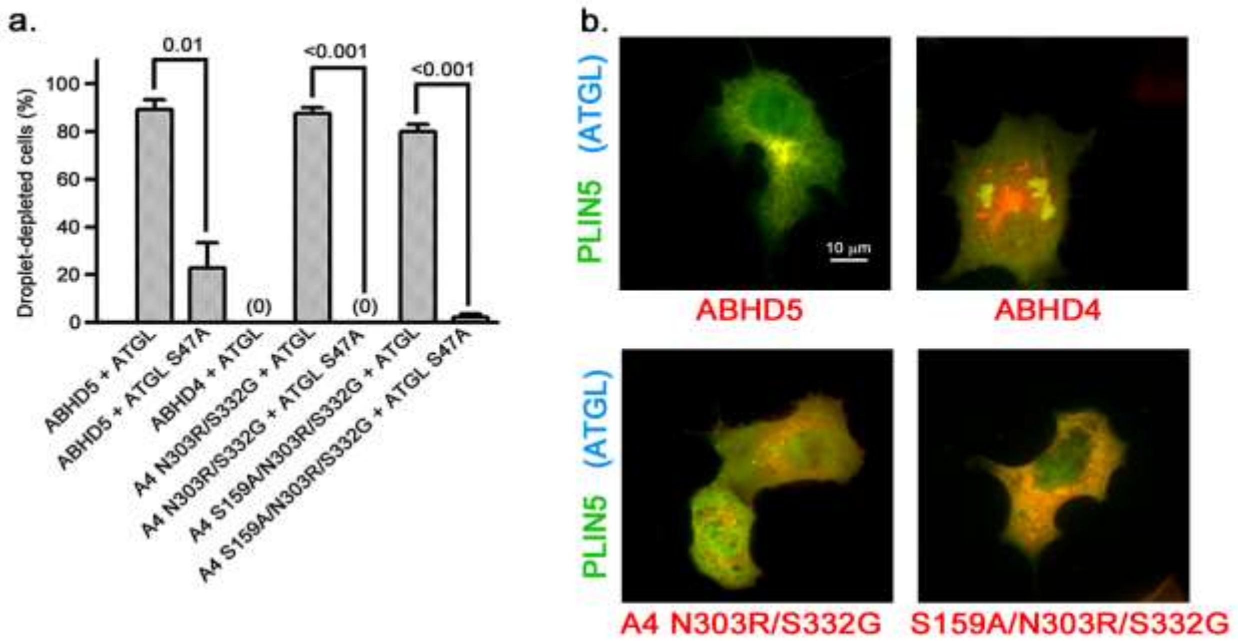

Supplementary Figure S1. ATGL activation by gain-of-function ABHD4 N303R/S332G is independent of ABHD4 hydrolase activity. **a)** Cos7 cells transfected with ABHD protein-mCherry, PLIN5-EYFP, and ATGL-CFP or ATGL S47A-CFP (lipase inactive) were lipid loaded overnight then fixed. Results are based on three independent experiments. **b)** Representative images of cells scored in **a)**. Scale bar = 10 µm.

Supplementary Figure S2

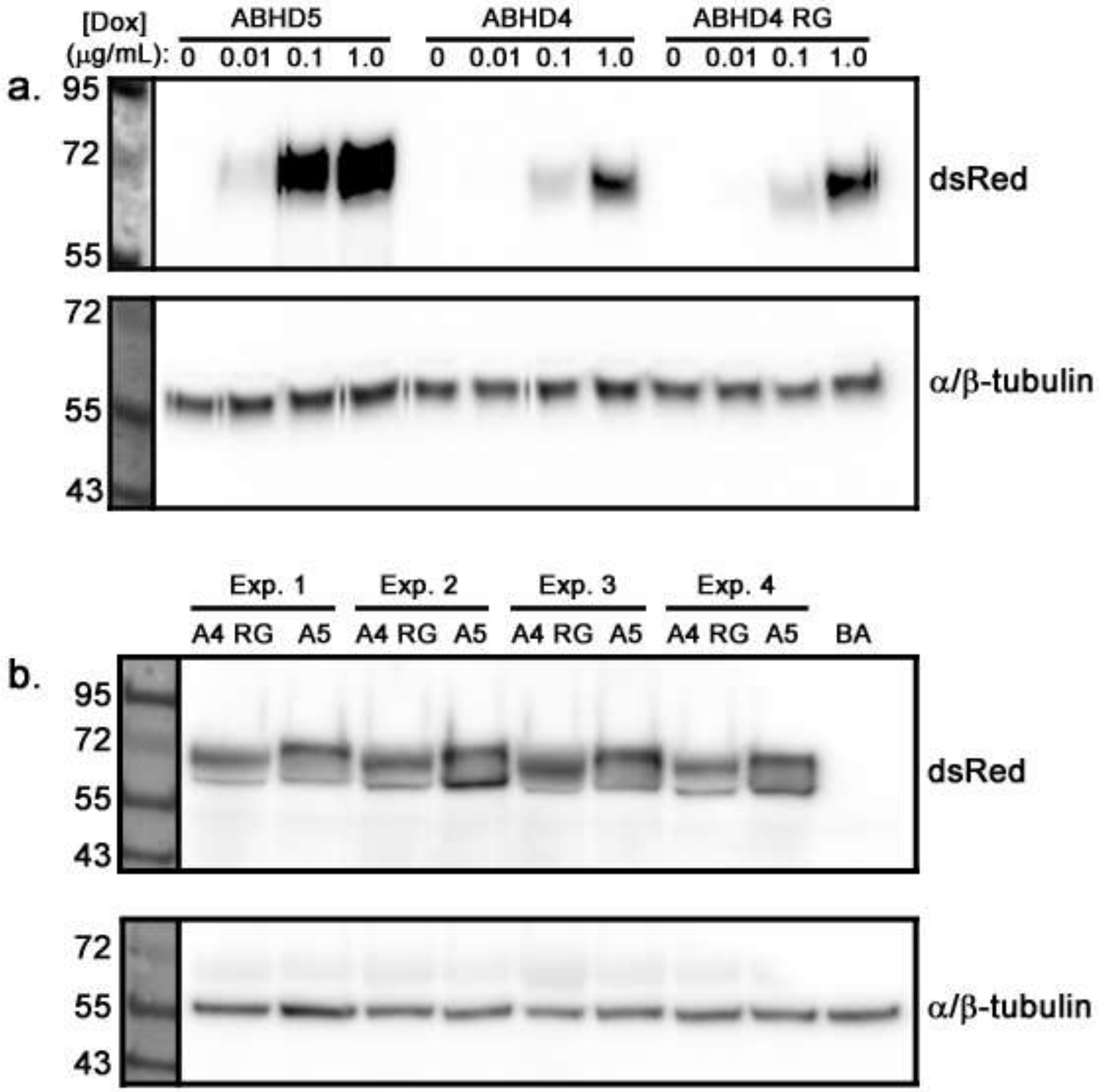

Supplementary Figure S2. Expression profile of re-expressed ABHD proteins in ABHD5-null BA cell lines. **a)** Representative immunoblot showing relative doxycycline-induced expression of ABHD5-mCherry, ABHD4-mCherry, and ABHD4 N303R/S332G-mCherry. This experiment was done twice with the same results. **b)** ABHD4 N303R/S332G-mCherry (A4 RG) and ABHD5-mCherry (A5) expression in BA cell lines from the four independent experiments used in figure 4c for comparing activity of the two proteins. ABHD4 N303R/S332G-mCherry expression was induced by 1 μg/ml doxycycline, while ABHD5-mCherry expression was induced by 0.0125 μg/mL doxycycline. BA, lysate from wild-type differentiated BA cells.
